# The Addition of Reishi and Lion’s Mane Mushroom Powder to Pasta Influences the Content of Bioactive Compounds and the Antioxidant, Potential Anti-Inflammatory, and Anticancer Properties of Pasta

**DOI:** 10.3390/antiox12030738

**Published:** 2023-03-17

**Authors:** Magdalena Szydłowska-Tutaj, Urszula Szymanowska, Krzysztof Tutaj, Dorota Domagała, Urszula Złotek

**Affiliations:** 1 Department of Biochemistry and Food Chemistry, University of Life Sciences, Skromna Str. 8, 20-704 Lublin, Poland; 2 PZZ Lubella GMW Sp. z o.o., Wrotkowska Str. 1, 20-469 Lublin, Poland; 3 Department of Biochemistry and Toxicology, University of Life Sciences, Akademicka Str. 13, 20-950 Lublin, Poland; 4 Department of Applied Mathematics and Computer Science, Faculty of Production Engineering, University of Life Sciences, Głęboka Str. 28, 20-612 Lublin, Poland

**Keywords:** mushrooms, pasta fortification, antioxidant activities, anti-inflammatory properties, anticancer properties

## Abstract

The influence of a 2.5% and 5% addition of dried Reishi and Lion’s Mane mushrooms on the content of bioactive compounds and some pro-health properties of pasta was studied. In samples subjected to gastrointestinal digestion, the content of phenolic compounds and the antioxidant, potential anti-inflammatory, and antiproliferative properties were significantly higher. The qualitative–quantitative analysis of phenolic compounds performed using the LC-MS/MS technique indicated that the Reishi-enriched pasta was characterized by a higher content of syringic (R2.5 sample), while pasta supplemented with Lion’ Mane had a higher content of vanillin in relation to the control pasta. In the case of ethanolic extracts, samples supplemented with the Reishi mushrooms (R5 sample) were characterized by higher ABTS antiradical properties and a reducing power while the sample supplemented with Lion’s Mane (L5 sample) had a higher ability to inhibit lipoxygenase in relation to the control sample. In conclusion, the results suggest that Reishi and Lion’s Mane mushroom powder can be used for the fortification of semolina pasta, conferring slightly healthier characteristics of the product.

## 1. Introduction

Fungi are among the most common organisms on Earth. They perform critical roles in the soil ecosystem by forming vast microscopic filamentous networks in symbiosis with the roots of most plants. The role of fungi in the ecosystem is connected with enhancing rock weathering and providing the nutrient supply to plants [1].

In addition to the numerous functions of fungi in the ecosystem, scientists from many research centers around the world conduct investigations of cultivated fungi as well as wild species given their documented biological activity with anticancer, antiviral, hepatoprotective, and immunomodulatory properties. Therefore, mushrooms can be used as a source of biotherapeutics and for the development of new drugs [2].

With their bioactive properties, mushrooms can also be used as ingredients of functional foods. They can serve as natural matrices of functional products and can be used as ingredients for the fortification of some food products [3].

Reishi (*Ganoderma lucidum*) and Lion’s Mane (*Hericium erinaceus*), among others, have been used as dried powders in research related to the fortification of pasta [4]. *Ganoderma lucidum,* known as Reishi, represents the group of polypores, i.e., mushrooms producing large fruiting bodies with pores or tubes on the underside. The Reishi mushroom is mainly used medicinally; so far, it has not been used in food due to its hard texture and bitter taste. It has been used to inhibit cancer growth and is well known in China, Japan, the USA, and other parts of the world as a source of drug components [2,5]. The main bioactive molecules that are present in Reishi mushrooms are peptidoglycans (Ganoderan A and B), glucans, triterpenes, heteropolysaccharides, and some phenolic compounds, mainly from the group of phenolic acids, i.e., protocatechuic acid, *p*-hydroxybenzoic acid, *p*-coumaric acid, and cinnamic acid [2,5]. This mushroom has documented healthy properties such as hypoglycemic, antioxidant, anticancer, antiviral (HIV-1), anti-allergic, anti-inflammatory, anti-hepatotoxic, cholesterol biosynthesis inhibitory, and antioxidant activities [2]. Currently, many products based on the Reishi mushroom are available in stores, for instance, powders, tea, dietary supplements, and cosmetics [5].

The other mushroom with both culinary and medicinal properties is *Hericium erinaceus*, commonly called Lion’s Mane [6]. It is widely consumed in Asian countries for its nutritional and health benefits and it has a long history of use in traditional Chinese medicine. This species can contribute to the mitigation, prevention, or even treatment of various serious diseases such as cancer, depression, diabetes, and neurodegenerative diseases [6]. The main active ingredients of Lion’s Mane are polysaccharides, terpenoids (especially hericenones and erinacines, which are specific for this species), steroids, alkaloids, lactones, and some phenolic compounds. These compounds are responsible for the anticancer, immunomodulating, and neuroprotective effects, they support the functioning of the nervous system at many levels (e.g., support of learning and memory processes and the stimulation of neurogenesis), and they support the circulatory system [7,8]. Additionally, products containing *Hericium erinaceus* are used in the treatment of Alzheimer’s disease.

The Lion’s Mane mushroom, apart from its health-promoting properties, is also valued for its taste, mainly in Asian countries [9].

So far, there are limited studies using Lion’s Mane for food fortification. Given the pro-health properties of this mushroom feature, we decided to make an attempt to use dried Lion’s Mane mushroom for pasta fortification.

In our previous work, we investigated the effect of the addition of Reishi and Lion’s Mane (in the amount of 2.5, 5, 7.5, and 10.0%) on the physicochemical, culinary, and organoleptic properties of semolina wheat pasta. Our results indicated that pasta with a 7.5% or 10% addition of these mushrooms had no satisfactory quality due to the negative sensory assessment, particularly the intensely bitter taste. In contrast, pasta enriched with Reishi and Lion’s Mane at the level of 2.5 and 5% were characterized by a high nutritional value and satisfactory culinary and organoleptic properties. [4].

The aim of this study was to evaluate the influence of a 2.5% and 5% addition of dried Reishi and Lion’s Mane mushrooms on some pro-health properties of pasta, especially reflected in the antioxidant, potentially anti-inflammatory, and anticancer activities.

## 2. Materials and Methods

### 2.1. Preparation of Pasta

The materials for pasta production were durum wheat semolina (obtained by milling durum wheat (*Triticum durum*) that was available from PZZ Lubella (Mill PZZ Lubella GMW Sp. z o.o. in Lublin, Poland), dried *Ganderma lucidum* (Reishi)*,* and *Hericium erinaceus* (Lion’s Mane). Mushrooms were purchased in food stores (NANGA Przemysław Figura, Złotów, Poland). Each mushroom had a quality certificate confirming its quality, expiry date, and country of origin. The centesimal compositions (g/100 g) of raw materials (semolina and added mushrooms) have been published in our previous study and they were as follows: Semolina: fat: 1.01, protein: 15.31, and carbohydrates: 68.09; Lion’s Mane (L): fat: 2.8, protein: 20.1, and carbohydrates: 23.9; Maitake (M): fat: 2.5 and protein: 15.0, carbohydrates [10].

Lion’s Mane and Reishi mushrooms were purchased as dried powder with a particle size of 80–100 mesh; next, the dried powder was sieved in our lab through with a 500 µm mesh sieve. The raw materials for the production of pasta (mushrooms and semolina) were stored in the laboratory in controlled temperature (max. 25 °C) with a relative humidity (max. 65%).

The tagliatelle pasta was prepared in reproducible laboratory conditions from semolina fortified with two types of dried mushrooms: Reishi (R) and Lion’s Mane (L) (2.5% (*w*/*w*); 5.0% (*w*/*w*)), as described previously [4]. The pasta was cooked at the optimal cooking time determined in our previous study [4]. Next, pasta was frozen, freeze-dried (LABCONCO, Kansas City, MO, USA), ground (MRC GRINDING MACHINE, SM-450, Holon, Israel), and used for further research.

### 2.2. Content of Some Bioactive Compounds

#### 2.2.1. Determination of Phenolic Compounds

##### Determination of Phenolic Acid Content (PAC)

The total content of phenolic acids was determined according to the Arnov method [11] and expressed as caffeic acid equivalent (CAE) in μg per g of dry weight (DW).

##### Determination of Total Flavonoid Content (TFC)

The total flavonoid content was determined according to the method described by Lamaison and Carnet (1990) [12]. It was calculated as quercetin equivalent (QE) in mg per g of dry weight (DW).

##### Determination of Total Phenolic Content (TPC)

The amount of total phenolics was determined using Folin–Ciocalteau reagent used for the determination all polyphenol fractions contained in the extract [13]. The amount of total phenolics was calculated as gallic acid equivalent (GAE) in mg per g DW.

Determination of phenolic compounds using spectrophotometric methods (PAC, TFC, and TPC) was made in ethanolic, PBS extracts, and in samples after in vitro digestion.

##### Qualitative–Quantitative Analysis of Phenolic Compounds Using the LC-MS/MS Technique

Briefly, phenolic compounds were released from the esterified form and from the cell wall by means of alkaline hydrolysis and then were extracted with ethyl acetate in acidic pH, as described by Żuchowski et al., with modifications [14]. The extraction was performed in triplicates. A total of 25 mg of the material was incubated with 500 µL of 0.2 M NaOH containing 1% of ascorbic acid as an antioxidant at 50 °C for 1 h. Prior to the hydrolysis, 25 μL of the internal standard (10 μg/mL of trans-cinnamic-d7 acid, Merck) was added. Acidification to pH 2 was carried out using 2 M HCl, and extraction was performed with 1 mL of ethyl acetate for 15 min at room temperature. Then, the extracts were centrifuged at 14,000× *g* for 10 min, and the organic layer was evaporated at 40 °C under a stream of nitrogen. The residue was reconstituted in 50 µL of water with methanol (9:1) and centrifuged at 14,000× *g*. The supernatant was transferred to a glass vial with insert for LC-MS/MS analysis.

The concentration of phenolic compounds was determined using a high performance liquid chromatograph (ExionLC AD, AB Sciex, Framingham, MA, USA) coupled with a mass spectrometer (QTRAP 6500+, AB Sciex, Framingham, MA, USA). Chromatographic separation was carried out on a Kinetex Biphenyl (100 mm × 3 mm, 2.6 µm) column (Phenomenex, Torrance, CA, USA).

Mobile phase: A—water with formic acid 0.1% (at the beginning 5%); B—acetonitrile with formic acid 0.1% (95%) and 0.4 mL/min flow. Gradient program: 0.0–1.0 min 5% B, 1.0–2.0 min 5–22% B, 2.0–5.0 min 28% B, 5.0–6.0 min hold 28% B, 6.0–10.0 min 28–70% B, 10.0–11.0 min 70–95% B, 11.0–12.0 min 95% B, 12.0–12.1 min 95–5% B, and 12.1–14.0 min 5% B. The injection volume was 5 µL. For detection, electrospray ionization (ESI) in the negative ion mode was used. Tandem mass spectrometry MS/MS was used for quantitative studies. The parameters of all molecules monitored with the MRM method (e.g., precursor (Q1), productions (Q2), collision energy (CE), and retention times) are listed in the Supplementary Materials (Supplementary File Table S1). The LC-MS/MS system was controlled using Analyst 1.7.2 software (AB Sciex, Framingham, MA, USA). SCIEX OS Version 2.1.6.59781 (AB Sciex, Framingham, MA, USA) was used for data processing.

In the case of ethanolic extracts, PBS, and digested samples, direct injections of extracts were analyzed with separation conditions identical to those in alkaline hydrolysis.

#### 2.2.2. Determination of Glucans

The contents of total alpha- and beta-glucans were determined using the K-YBGL β-glucan Assay Kit (Yeast and Mushrooms) (Megazyme, Bray, Ireland) according to the manufacturer’s instructions. The dried and milled pasta samples (100 mg) were used for the extraction procedure. The results were calculated according to the manufacturer’s instructions and expressed as g/100 g DW.

### 2.3. Preparation of Extracts

#### 2.3.1. Ethanolic Extracts

Ethanolic extracts were prepared using sonication (1g DW in 10 mL of 50% ethanol, sonication at 30 °C for 1 h, and then centrifugation at 9000× *g* for 30 min).

#### 2.3.2. PBS Extracts

For preparation of buffer extracts (PBS), a freeze-dried and grounded sample (1 g) was homogenized, extracted for 60 min with 10 mL of PBS buffer (phosphate-buffered saline, pH 7.4), and centrifuged at 9000× *g* for 20 min.

#### 2.3.3. In Vitro Digestion

In vitro digestion was performed according to the procedure described by Minekus et al. [15], with slight modifications, proposed by Sęczyk et al. [16].

Before the first step of digestion, samples (1 g) were homogenized with 1 mL of distilled water. After all phases of digestion (gastrointestinal digested samples—GID), the samples were centrifuged (15 min, 6900× *g*), and the supernatants were used for analysis. The gastric phase (gastro digested samples—GD) was collected for determination of potential anticancer properties.

### 2.4. Antioxidant Activities

Determination of antioxidant activities was made in ethanolic, PBS extracts, and in samples after in vitro digestion.

#### 2.4.1. Free Radical Scavenging Assays

Free radical scavenging activity was measured using 1,1-diphenyl-2-picrylhydrazyl (DPPH) according to Brand-Williams, Cuvelier, and Berset (1995) [17] and 2,2′-azino-bis(3-ethylbenzothiazoline-6-sulphonic acid (ABTS^+•^), according to Re et al. (1999) [18], as a source of free radicals. The antioxidant activity was related to Trolox (an analogue of vitamin E) and expressed as mg of Trolox per gram of dry weight (DW).

#### 2.4.2. Ferric-Reducing Antioxidant Power

Ferric-reducing antioxidant power (RP) was determined according to the methods described by Oyaizu [19]. Reducing power was expressed as a Trolox equivalent (TE) in mg of Trolox per gram of dry weight (DW).

#### 2.4.3. Chelating Power

Chelating power (CHP) was determined using the method developed by Guo et al. [20]. The chelating power was expressed as an EDTA equivalent in µg EDTA per g of dry weight (DW).

### 2.5. Determination of Potential Anti-Inflammatory Properties

Determination of potential anti-inflammatory properties was made in ethanolic, PBS extracts, and in samples after in vitro digestion.

#### 2.5.1. LOX Inhibitory Activity

The impact of extracts from the control sample and pastas supplemented with dried mushrooms on the lipoxygenase (LOX) activity was measured spectrophotometrically according to the method described by Szymanowska et al. [21], and adapted to the BioTek Microplate Reader (Winooski, VT, USA). One unit of LOX activity was defined as an increase in absorbance of 0.001 per minute at 234 nm. The corresponding control contained the same concentration of the enzyme in the absence of the inhibitor. An extract concentration (mgDW/mL) providing 50% inhibition (EC50) was obtained by plotting the inhibition percentage against sample concentrations.

#### 2.5.2. COX2 Inhibitory Activity

The impact of the analyzed extracts on cyclooxygenase-2 activity was determined spectrophotometrically at 590 nm by measuring the activity of the COX peroxidase subunit using NNN′N′-tetramethyl-p-phenylenediamine (TMPDA) as an electron donor, with the use of the COX Activity Assay kit from Cayman Chemical (Cayman Chemical, MI, USA). COX activity was determined according to the instructions provided with the kit. An extract concentration (mg DW/mL) providing 50% inhibition (EC50) was obtained by plotting the inhibition percentage against sample concentrations.

### 2.6. Determination of Potential Anticancer Properties

The potential anticancer properties of the studied pastas were tested using two cancer cell lines: AGS: Human Caucasian gastric adenocarcinoma (ECACC No. 89090402) and HT 29: Human Caucasian colon adenocarcinoma (ATCC HTB-38). The cells (0.5 × 106 cells/mL) were seeded in 96-well plates and incubated in an air atmosphere humidified with 5% of CO_2_ at 37 °C for 24 h. The growth medium consisted of DMEM F12 (for AGS) or RPMI 1640 medium (for HT29), 10% FBS (heat-inactivated fetal bovine serum), 2 mM L-glutamine, and a 1% antibiotic-antimycotic solution (Sigma-Aldrich, Poznań, Poland). One day after cell seeding, the medium was replaced by samples subjected to gastric (GD samples) or gastrointestinal digestion (GID samples). After 24 h incubation at 37 °C, the cell lines were exposed to various amounts of gastric (GD) or gastrointestinal (GID) samples in DMEM F12 and RPMI, respectively, and were further incubated for 24 h. The final concentration of gastric and gastrointestinal fluids did not affect cell viability. Then, the WST-1 assay kit (BioVision, Inc., San Francisco, CA, USA) was used for cytotoxicity evaluation according to the manufacturer’s procedure.

The cytotoxicity was determined as a percentage of living cells in comparison to the control. The results were evaluated by determination of the EC50 (effective concentration of 50% cell viability) values, which were expressed as mg DW/mL. Each experiment was repeated three times.

### 2.7. Statistical Analysis

All determinations were performed in triplicates unless otherwise stated. Statistical analysis was performed using Statistica ver. 13.3 software. The non-parametric Kruskal–Wallis test was used to examine whether there were any statistically significant differences between groups at the significance level *p* ≤ 0.05. Homogenous groups were determined by the Dunn test, and data were reported as mean ± standard deviation.

## 3. Results

### 3.1. Content of Bioactive Compounds

The contents of total phenolic compounds, total flavonoids, and phenolic acids in the pasta with the 2.5% and 5% addition of dried Reishi and Lion’s Mane mushrooms after ethanol and PBS extraction and after simulated digestion (gastrointestinal digested samples—GID) are presented in Table 1. The total phenolic content (TPC) in Reishi-fortified samples achieved the highest value of 5.44 mg/gDW in the R5 sample after gastrointestinal digestion (Table 1).

A similar trend was observed in the total flavonoid content (TFC), i.e., an increase in the dried Reishi mushroom dose was accompanied by an increase in the TFC in all extracts. A similar trend was observed in samples with the addition of dried Lion’s Mane mushroom, except for the sample subjected to digestion, where a higher value was observed for the L2.5 sample. The contents of phenolic acid (PAC) in all the extracts were similar (no statistically significant differences between the tested samples were found; *p* = 0.08, 0.1319, and 0.296 for ethanolic, PBS, and GID extracts, respectively), regardless of the concentration of the dried mushrooms used; they also oscillated around the value noted in the control sample.

Using the LC-MS/MS technique, 15 compounds were identified in the tested samples, but the content of some of these compounds was below the limit of determination of the lowest calibration point (Table 2). Ferulic acid was the dominant phenolic acid in all the samples (content between 140.91 and 154.13 µg/g DW). It should be noted that, in some cases, the fortification with the studied mushrooms had an influence on the phenolic compound content. The fortification with Reishi resulted in higher amounts of syringic acid (Table 2). In turn, the addition of the Lion’s Mane dried powder resulted in a higher content of vanillin in relation to the control pasta (Table 2).

The identification of phenolic compounds in ethanolic, PBS, and GID extracts confirmed the presence of compounds in the raw materials that were also in the extracts used for the study (Table S2). In the ethanolic extracts of pasta enriched with Reishi, a significantly higher content of the following compounds was noted compared to the control: 3,4-dihydroxybenzoic acid (R5 sample) and caffeic acid (R2.5 and R5 samples). In the case of PBS extracts, a statistically significant difference (*p* = 0.0097) was noted only for the content of syringic acid—extracts from pasta enriched with Reishi (R5) showed the highest content of this phenolic acid. In the samples after simulated digestion, statistically significant differences were noted only in the contents of 3,4-dihydroxybenzoic acid (which was detected only in R2.5 and R5 samples) syringic acid, and sinapic acids (*p* = 0.0112 and 0.0122, respectively)—a higher content compared to the control was observed for the R5 and R2.5 samples, respectively.

Table 3 summarizes the content of glucans in the studied pasta. In all the samples, the content of the beta-glucan fraction was higher than that of the alpha-glucans; surprisingly, the addition of the mushrooms did not cause statistically significant differences in the content of these components (Table 3).

### 3.2. Antioxidant Activity

The antioxidant activities measured by DPPH and ABTS radical scavenging activity, chelating power, and reducing power of the pasta with the 2.5% and 5% addition of dried Reishi and Lion’s Mane mushrooms after ethanol and PBS extraction and after gastrointestinal digestion are presented in Table 4.

The ABTS scavenging ability of the ETOH and PBS extracts was significantly lower (from 0.68 mg TE/gDW in sample C extracted with ETOH to 1.35 mg TE/gDW for sample R2.5 extracted with PBS) than in the in vitro digested samples (12.14–12.49 mg TE/gDW). It should be noted that the ethanolic extract samples supplemented with Reishi mushrooms (R5 sample) were characterized by statistically significantly higher antiradical activity (against ABTS) than the control sample. In the GID samples, in both cases (Reishi and Lion’s Mane addition), the increase in the amount of the dried mushrooms was accompanied by an increase in the value of antiradical activity against ABTS. In the case of the antiradical activity against DPPH, the highest activity was also observed for GID samples with values ranging from 3.28 to 4.04 mg TE/gDW. However, there were no significant differences between the study groups. The lowest activity or no activity was observed for the ETOH samples. It should be noted that only the ethanolic extract from the R2.5 and R5 samples exhibited antiradical activity against DPPH.

In the case of the chelating power, the PBS extracts of all samples (control and with mushrooms addition) as well as the R5 and L5 samples after ethanolic extraction showed no activity. The highest CHP activity, i.e., 217.94 mgEDTA/gDW, was found in the control sample after simulated digestion.

The values of the reducing power of the analyzed samples ranged from 0.19 mgTE/gDW for the control ethanolic sample to 1.52 mgTE/gDW for the GID R5 sample. The highest values were found in the samples subjected to digestion. In the case of the ETOH and PBS samples, the lowest RP was detected in the control sample. A significant increase in the value of RP was found in the samples supplemented with 5% of Reishi (*p* = 0.0016 and 0.0079, for ETOH and PBS extract, respectively).

### 3.3. Potential Anti-Inflammatory Properties

The ability of the studied samples to inhibit enzymes involved in inflammation, i.e., lipoxygenase and cyclooxygenase 2, is presented in Table 5. The ethanolic extracts from pasta fortified with Lion’s Mane mushrooms (L5 sample) showed a statistically significantly higher ability to inhibit LOX (the EC50 values were 0.15 mg/mL) than the control (EC50 = 0.52 mg/mL)—*p* = 0.0349. It should also be noted that the PBS extract from the control sample showed no ability to inhibit lipoxygenase activity, while the PBS extracts from pasta fortified with the studied mushrooms were characterized by the ability to inhibit LOX at the EC50 level ranging from 0.42 mg/mL to 0.34 mg/mL (Table 5). The GID extracts were characterized by a significantly higher LOX inhibition capacity compared to the ETOH and PBS extracts, but the activity of the GID samples supplemented with the mushrooms was not statistically significantly different from the control sample.

The PBS extracts from all the studied pasta (control and fortified with Reishi and Lion’s Mane) did not show COX-2 inhibitory activity (Table 5). However, the ethanol extracts and the in vitro digested samples showed a significant ability to inhibit COX-2; similarly to LOX inhibition, the GID samples showed greater activity compared to the ethanol extracts. However, the fortification with the studied mushrooms did not result in a statistically significant increase in this activity.

### 3.4. Potential Anticancer Properties

The samples from the studied pastas subjected to gastric (GD) and gastrointestinal digestion (GID) showed dose-dependent cytotoxic activity against the AGS and HT 29 cancer cell lines, respectively (Table 6). The GID samples showed higher antiproliferative activity (against the HT 29 cancer line; EC50 = 0.07–0.08 mg/mL) than the GD samples (against the AGS cancer line; EC50 = 0.15–0.28 mg/mL). However, there were no significant differences between the study groups.

## 4. Discussion

Mushrooms can be defined as large fungi that are capable of forming hard fungal tissue or large fleshy masses [22].

Mushrooms are consumed because they not only contain nutrients, e.g., protein, carbohydrates, dietary fiber, vitamins, and minerals, but they also possess documented pharmacological properties, such as immunomodulatory, anticancer, and even dementia-preventing activities. The biological properties are associated with many bioactive compounds that are abundant in mushrooms, e.g., phenolics, terpenoids, polysaccharides, glucans, and lectins [23].

Therefore, with their nutritional value, pro-health properties, and sensory and textural traits, mushrooms can be used in the food industry as possible substitutes for some ingredients or as food additives [24].

In the food industry, mushrooms can be used in a direct way (as an ingredient or additive to food products) or in an indirect way (as a source of fermentation) in food products [25].

There are some reports on the use of edible mushrooms as food ingredients or additives [24,26]. Among others, there are attempts to add mushrooms to flour-based products, such as bread, muffins, or pasta [24,27].

However, the addition of ingredients without gluten proteins or high-fiber ingredients (for example, mushroom powder) as replacements for commonly used flour may change some dough properties. Therefore, in our previous study, we selected recipes for the addition of such ingredients to flour products in terms of satisfactory physicochemical and sensory properties before assessment of their pro-health activities [4].

Our previous study on the culinary and sensory properties of durum wheat pasta enriched with dried mushrooms indicates that the optimal level of supplementation with dried Reishi and Lion’s Mane mushrooms as a semolina flour replacer is 2.5 and 5% [4].

The use of the Lion’s Mane as well as Reishi mushrooms in the production of food products has so far been limited [27,28].

The addition of mushroom powder to flour-based products such as breads, cakes, biscuits, or pasta has been studied recently, but mainly in the context of nutrition and consumer quality of obtained products [4,29]. There are many studies confirming its health-promoting properties, which are attributed to the bioactive compounds contained in this mushroom, i.e., triterpenoids, polyphenols, and biologically active polysaccharides [5,30]

The beneficial biological activity of Reishi and Lion’s Mane has aroused research interest in using these mushrooms as food additives. However, so far, there are scarce data on the effect of the addition of mushrooms to flour-based products on health-promoting properties.

Hence, the influence of a 2.5% and 5% addition of dried Reishi and Lion’s Mane mushrooms on the content of bioactive compounds and some pro-health properties of pasta was analyzed in the present study.

In Veljovic et al. [31] study, the glucans content in Reishi mushrooms was determined using the same protocol as in the present study. This research indicated that the content of total glucans and β-glucans in Reishi was 9.44–18.55 g/100 g and 8.90–15.64 g/100 g, respectively, depending on the extraction time and particle size.

Whereas, based on the literature data, glucan content in Lion’s Mane varies slightly depending on the procedure used; the procedure used in the present study regarding the content of total glucans and β-glucans, determined by McCleary and Draga, was 37.1 g/100 g and 33.9 g/100 g, respectively [32].

Surprisingly, the fortification with the dried Reishi and Lion’s Mane mushrooms did not cause statistically significant differences in the content of glucans in the studied pasta (Table 3). These results are difficult to explain; perhaps the addition of dried mushrooms used in this study turned out to be too small.

Polyphenols are bioactive compounds with well-documented pro-health properties. Based on the literature data, the content of phenolic compounds in mushrooms depends on the kind of extraction; for the TPC content in Reishi, it varies from 86 to 139 mg/g and for the ethanolic extract, it varies from 33 to 47 mg/g for water extract [31], while in Lion’s Mane, according to Gąsecka et al. [33], the TPC content was 17.10 mg/g. Phenolic acids are the main fraction of this group of compounds present in the mushrooms used in the present study [5,34].

Gallic, *p*-coumaric, and *p*-hydroxybenzoic acids are the most often described phenolic acids in Lion’s Mane extracts [34,35], while protocatechuic, *p*-hydroxybenzoic, *p*-coumaric, syringic, and cinnamic acids are most commonly identified in Reishi extracts [5,36]. There are also reports of detection of protocatechuic, 4-hydroxybenzoic, chlorogenic, vanillic, caffeic, syringic, *p*-coumaric, ferulic, sinapic, salicylic, gallic, and trans-cinnamic acids as well as rutin, quercetin, and kaempferol in Lion’s Mane [33,37]. In a study conducted by Gąsecka et al. [33], phenolic compounds were also identified in an extract from *Ganoderma lucidum*, namely 4-hydroxybenzoic, chlorogenic, vanillic, caffeic, sinapic, and trans-cinnamic acids, rutin, quercetin, and kaempferol.

Based on their research, Lu et al. [38] suggested that pasta could be a good medium to add antioxidant and bioactive compounds to in order to enhance human nutrition. There are some reports on the use of phenolics-rich powder or extracts from plants or mushrooms as a strategy in the production of functional pasta [39]. Mushrooms can also be a valuable source of polyphenols in the diet. In a study conducted by Lu et al. [40], the contribution of mushroom powder supplementation to the phenolic content in pasta was studied. In this study, powder from white button, shiitake, and porcini mushrooms was added to pasta in three amounts: 5, 10, and 15%. All the mushroom powder-fortified pastas were characterized by a significantly higher content of TPC than that in the control sample, except for the 5% and 10% shiitake mushroom pasta variants. A similar effect was obtained in the present study. The qualitative–quantitative analysis of phenolic compounds indicated that the ethanolic extracts of the Reishi-enriched pasta were characterized by a higher content of 3,4-dihydroxybenzoic acid and caffeic acid in comparison to the control pasta (Table S2).

The fortification of food with bioactive compound-rich ingredients should result in the increased pro-health potential of the obtained products. Lu et al. [40] studied the effect of the addition of 5, 10, and 15% of white button, shiitake, and porcini mushrooms on the antioxidant properties (DPPH and ORAC methods) of pasta. An increase in the DPPH scavenging activity was exhibited by pasta supplemented with 10% and 15% of white button mushrooms. This effect was achieved in the shiitake and porcini supplementation variants only in the case of the 15% addition. In turn, the antioxidant activity determined with the ORAC method was higher in all pasta variants that were enriched with the tested mushrooms in comparison to the control pasta [40]. Additionally, in a study performed by Ibrahim et al. [41], the total phenolic content, total flavonoid content, DPPH scavenging activity, and FRAP values of waffles, breadsticks, and salad cream supplemented with *Oyster* mushroom powder were higher than in the control sample.

In our study, the fortification of pasta with Reishi caused an increase in antioxidant activity, mainly in the ethanolic extracts (Table 4). Most importantly, samples subjected to gastrointestinal digestion were characterized by a significantly higher content of polyphenolic compounds as well as antioxidant and potentially anti-inflammatory potential than the other ones (Table 1, Table 4, and Table 5). Similar results were obtained by Sęczyk et al. [16] in their study on pasta fortified with parsley leaves.

Dong et al. [42] showed strong correlations between the DPPH and FRAP antioxidant activities and polyphenolic content in Reishi mushrooms. Similarly, Saltarelli et al. [43] found a positive correlation between the content of phenolic compounds in Reishi extracts and antioxidant properties, i.e., DPPH and chelating power, as well as lipoxygenase inhibition.

Phenolic compounds as well as other bioactive compounds, e.g., β-glucans, glycoproteins, or triterpenes, contained in mushrooms may also have anti-inflammatory effects. As evidenced by Taofiq et al. [44], the anti-inflammatory properties are mainly ascribed to phenolic acids (especially *p*-hydroxybenzoic, *p*-coumaric, and cinnamic acids) and their derivatives contained in mushrooms.

Among the anti-inflammatory mechanisms of action such as the inhibition of transcription factors linked to inflammation and pro-inflammatory cytokines, the inhibition of pro-inflammatory enzymes (lipoxygenase, cyclooxygenase-2, and inducible nitric oxide synthase) should be highlighted [44,45]. The ability of the extracts from the studied pastas to inhibit LOX and COX-2 was determined in the present work (Table 5). The ethanol extracts and the in vitro digested samples from all the pasta variants showed a significant ability to inhibit the activity of these pro-inflammatory enzymes. What is noteworthy is that the samples subjected to simulated digestion exhibited significantly higher potential anti-inflammatory activity than the PBS and ethanol extracts. These differences are most likely related to the differences in the content of phenolic compounds in the extracts—after in vitro digestion, more phenolic compounds are found in the sample because they are released as a result of the action of digestive enzymes and simulated conditions of the digestive tract (mainly pH) [14]. Similar results have been seen in other studies using simulated digestion [46,47]. Additionally, in the case of the ethanolic extracts, samples from pasta fortified with Lion’s Mane mushrooms (L5 sample) showed a statistically significantly higher ability to inhibit LOX in comparison to the control sample (Table 5).

Among the health-promoting properties of Reishi and Lion’s Mane mushrooms, their documented anticancer properties deserve special attention. The antitumor activity exhibited by *Ganoderma lucidum* is achieved via the induction of programmed cell death, as reported by many studies [48,49,50]. A methanolic extract of *Ganoderma lucidum* fruiting bodies prevented the growth of a human gastric tumor cell line, as reported by Reis et al. and Oliveira et al. [49,50]. Additionally, research conducted by Kolniak-Ostek et al. [30] confirmed the potential anticancer activity of *Ganoderma lucidum* bioactive compounds against breast and colorectal cancer.

Some reports have also indicated the anticancer properties of extracts from *Hericium erinaceus* via the reduction of cell proliferation, induction of apoptosis in cancer cells [51], and inhibition of the migration and invasion of cancer cells by reduction of the expression of matrix metalloproteinases MMP-2 and MMP-9 in cancer cells [52].

In their publication, Kolniak-Ostek et al. [30] suggested that a significant part of the antiproliferative activity of *Ganoderma lucidum* extract toward cancer cells might be attributed to the high content of phenolic compounds (especially resveratrol and apigenin). Additionally, Sęczyk et al. [16] indicated that the anticancer activity (against breast carcinoma cells) of pasta fortified with parsley leaves was correlated with the phenolic content, but a relatively high concentration of the extract caused a cytostatic effect in their research. In the present study, not very high concentrations of the samples of the digested pasta (especially in the case of gastrointestinal digestion) were necessary to obtain a cytostatic effect on carcinoma cells (Table 6).

## 5. Conclusions

In conclusion, the results obtained in the present study suggest that Reishi and Lion’s Mane mushroom powder can be used for the fortification of semolina pasta, conferring slightly healthier characteristics connected with the phenolic compound content, mainly in terms of the antioxidant and potentially anti-inflammatory properties of the product (determined mainly in ethanolic and PBS extracts). However, further research is needed because the potentially bioavailable fraction of bioactive compounds contained in the fortified pasta samples after simulated digestion did not show (with some exceptions) significant differences regarding the tested biological properties.

## Figures and Tables

**Table 1:** Phenolic compound content in pasta supplemented with dried Reishi and Lion’s Mane mushrooms.

Samples	PAC (µg/g DW)			TFC (mg/g DW)			TPC (mg/g DW)		
	ETOH	PBS	GID	ETOH	PBS	GID	ETOH	PBS	GID
C	0.17 ± 0.01 ^a^	1.33 ± 0.02 ^a^	3.11 ± 0.33 ^a^	0.17 ± 0.12 ^a^	0.04 ± 0.04 ^a^	4.45 ± 0.08 ^a^	0.60 ± 0.06 ^a^	0.55 ± 0.10 ^a^	5.27 ± 0.30 ^a^
R2.5	0.20 ± 0.03 ^a^	1.36 ± 0.07 ^a^	3.07 ± 0.30 ^a^	0.48 ± 0.13 ^a^	0.38 ± 0.08 ^a^	4.19 ± 0.28 ^a^	0.77 ± 0.21 ^a^	0.81 ± 0.05 ^a^	5.35 ± 0.70 ^a^
R5	0.20 ± 0.01 ^a^	1.41 ± 0.07 ^a^	3.21 ± 0.08 ^a^	0.82 ± 0.35 ^a^	0.64 ± 0.13 ^a^	5.08 ± 0.38 ^a^	1.01 ± 0.18 ^a^	1.08 ± 0.11 ^a^	5.45 ± 0.83 ^a^
L2.5	0.17 ± 0.01 ^a^	1.29 ± 0.08 ^a^	3.13 ± 0.06 ^a^	0.73 ± 0.62 ^a^	0.04 ± 0.02 ^a^	4.84 ± 0.44 ^a^	0.67 ± 0.23 ^a^	0.66 ± 0.07 ^a^	5.37 ± 0.47 ^a^
L5	0.18 ± 0.01 ^a^	1.28 ± 0.07 ^a^	3.00 ± 0.02 ^a^	0.80 ± 0.47 ^a^	0.10 ± 0.07 ^a^	4.44 ± 0.56 ^a^	0.64 ± 0.09 ^a^	0.70 ± 0.12 ^a^	5.20 ± 0.42 ^a^
*p-value*	*0.0928*	*0.4169*	*0.1329*	*0.1361*	*0.0708*	*0.0994*	*0.1870*	*0.0617*	*0.9339*

C—control (pasta from semolina); R2.5-5—pasta from semolina flour fortified with 2.5–5% of Reishi mushroom powder; L2.5-5—pasta from semolina flour fortified with 2.5–5% of Lion’s Mane mushroom powder; PAC—phenolic acid content; TFC—total flavonoid content; TPC—total phenolic content. Means with different superscripts within a column are significantly different at *p* ≤ 0.05.

**Table 2:** Qualitative–quantitative analysis of phenolic compounds using the LC-MS/MS technique.

Name of Polyphenolic Compound	Polyphenolic Compound (μg/g DW)														
	3,4-Dihydroxy -Benzoic Acid	Caffeic Acid	Syringic Acid	Daidzin	Rutin	Ellagic Acid	*p*-Coumaric Acid	Salicylic Acid	Vanillin	Ferulic Acid	Sinapic Acid	Rosmarinic Acid	t-Cinnamic Acid	Genistein	Naringenin
C	<0.5	<0.5	<0.5	n.d	<0.5	n.d	1.67 ± 0.03 ^ab^	<0.5	0.56 ± 0.11 ^a^	154.13 ± 13.21 ^a^	27.59 ± 1.24 ^ab^	<0.5	<0.5	n.d	n.d
R2.5	<0.5	<0.5	0.87 ± 0.02 ^a^	<0.5	<0.5	<0.5	1.96 ± 0.13 ^ab^	<0.5	0.70 ± 0.03 ^ab^	140.91 ± 3.88 ^a^	29.01 ± 0.69 ^ab^	<0.5	<0.5	<0.5	<0.5
R5	<0.5	<0.5	1.55 ± 0.05 ^a^	<0.5	< 0.5	<0.5	1.60 ± 0.23 ^a^	<0.5	0.74 ± 0.15 ^ab^	142.10 ± 7.19 ^a^	31.32 ± 0.92 ^ab^	<0.5	<0.5	<0.5	<0.5
L2.5	<0.5	<0.5	< 0.5	n.d	<0.5	<0.5	2.14 ± 0.08 ^ab^	<0.5	0.72 ± 0.10 ^ab^	145.70 ± 7.71 ^a^	25.65 ± 0.90 ^a^	<0.5	<0.5	n.d	n.d
L5	<0.5	<0.5	< 0.5	n.d.	< 0.5	<0.5	2.55 ± 0.15 ^b^	<0.5	0.89 ± 0.07 ^b^	158.29 ± 9.35 ^a^	33.03 ± 1.99 ^b^	<0.5	<0.5	n.d	n.d
*p-value*	-	-	*0.0809*	*-*	*-*	*-*	*0.0164*	*-*	*0.0349*	*0.1824*	*0.0125*	-	-	-	-

C—control (pasta from semolina); R2.5-5—pasta from semolina flour fortified with 2.5–5% of Reishi mushroom powder; L2.5-5—pasta from semolina flour fortified with 2.5–5% of Lion’s Mane mushroom powder, <0.5 below the limit of determination of the lowest calibration point; n.d.—not detected. Means with different superscripts (a,b) within a column are significantly different at *p* ≤ 0.05.

**Table 3:** Glucan content in pasta supplemented with dried Reishi and Lion’s Mane mushrooms.

Sample	Total Glucan (g/100 g)	α-Glucan (g/100 g)	β-Glucan (g/100 g)
C	0.28 ± 0.01 ^a^	0.10 ± 0.01 ^ab^	0.18 ± 0.01 ^a^
R2.5	0.29 ± 0.01 ^a^	0.10 ± 0.01 ^ab^	0.19 ± 0.01 ^a^
R5	0.28 ± 0.01 ^a^	0.10 ± 0.01 ^ab^	0.18 ± 0.01 ^a^
L2.5	0.28 ± 0.01 ^a^	0.10 ± 0.01 ^a^	0.18 ± 0.01 ^a^
L5	0.28 ± 0.01 ^a^	0.09 ± 0.01 ^b^	0.18 ± 0.01 ^a^
*p-value*	*0.4846*	*0.0248*	*0.8148*

C—control (pasta from semolina); R2.5-5—pasta from semolina flour fortified with 2.5–5% of Reishi mushroom powder; L2.5-5—pasta from semolina flour fortified with 2.5–5% of Lion’s Mane mushroom powder. Means with different superscripts within a column are significantly different at *p* ≤ 0.05.

**Table 4:** Antioxidant properties of pasta supplemented with dried Reishi and Lion’s Mane mushrooms.

Samples	ABTS (mg TE/gDW)			DPPH (mg TE/gDW)			CHP (mg EDTA/gDW)			RP (mgTE/gDW)		
	ETOH	PBS	GID	ETOH	PBS	GID	ETOH	PBS	GID	ETOH	PBS	GID
C	0.68 ± 0.04 ^b^	1.15 ± 0.11 ^ab^	12.47 ± 1.34 ^a^	n.a.	0.25 ± 0.01 ^a^	4.05 ± 0.08 ^a^	0.26 ± 0.16 ^ab^	n.a.	217.94 ± 53.82 ^a^	0.20 ± 0.02 ^a^	0.20 ± 0.08 ^a^	1.13 ± 0.06 ^a^
R2.5	0.88 ± 0.04 ^ab^	1.35 ± 0.12 ^a^	12.15 ± 0.37 ^a^	0.06 ± 0.05 ^a^	0.09 ± 0.10 ^a^	3.71 ± 0.35 ^a^	0.36 ± 0.11 ^a^	n.a.	92.53 ± 5.65 ^ab^	0.45 ± 0.02 ^ab^	0.38 ± 0.07 ^ab^	1.12 ± 0.14 ^a^
R5	1.04 ± 0.08 ^a^	0.91 ± 0.09 ^b^	12.26 ± 0.23 ^a^	0.08 ± 0.04 ^a^	0.25 ± 0.11 ^a^	3.81 ± 0.34 ^a^	n.a.	n.a.	77.41 ± 66.90 ^b^	0.66 ± 0.03 ^b^	0.73 ± 0.16 ^b^	1.52 ± 0.24 ^a^
L2.5	0.68 ± 0.10 ^b^	0.95 ± 0.08 ^b^	12.40 ± 0.20 ^a^	n.a.	0.16 ± 0.04 ^a^	3.44 ± 0.71 ^a^	0.14 ± 0.02 ^b^	n.a.	115.50 ± 45.93 ^ab^	0.25 ± 0.04 ^a^	0.21 ± 0.07 ^a^	1.06 ± 0.19 ^a^
L5	0.71 ± 0.09 ^ab^	1.02 ± 0.04 ^ab^	12.49 ± 0.41 ^a^	n.a.	0.20 ± 0.17 ^a^	3.28 ± 0.80 ^a^	n.a.	n.a.	125.47 ± 17.53 ^ab^	0.30 ± 0.05 ^ab^	0.21 ± 0.09 ^ab^	1.17 ± 0.09 ^a^
*p-value*	*0.005*	*0.0036*	*0.6939*	*0.4705*	*0.1463*	*0.1050*	*0.0194*	*-*	*0.0265*	*0.0016*	*0.0079*	*0.0911*

C—control (pasta from semolina); R2.5-5—pasta from semolina flour fortified with 2.5–5% of Reishi mushroom powder; L2.5-5—pasta from semolina flour fortified with 2.5–5% of Lion’s Mane mushroom powder; ABTS—radical scavenging ability against ABTS; DPPH—radical scavenging ability against DPPH; CHP—chelating power; RP-reducing power; n.a.—no activity. Means with different superscripts within a column are significantly different at *p* ≤ 0.05.

**Table 5:** Lipoxygenase and cyclooxygenase-2 inhibitory activity of extracts from pasta supplemented with dried Reishi and Lion’s Mane mushrooms.

Samples	LOXI (EC50 mg/mL)			COX2I (EC50 mg/mL)		
	ETOH	PBS	GID	ETOH	PBS	GID
C	0.52 ± 0.13 ^a^	n.a.	0.10 ± 0.01 ^a^	0.25 ± 0.05 ^a^	n.a.	0.22 ± 0.03 ^a^
R2.5	0.19 ± 0.04 ^ab^	0.35 ± 0.08 ^a^	0.10 ± 0.01 ^a^	0.52 ± 0.20 ^a^	n.a.	0.21 ± 0.05 ^a^
R5	0.18 ± 0.04 ^ab^	0.37 ± 0.04 ^a^	0.10 ± 0.01 ^a^	0.24 ± 0.03 ^a^	n.a.	0.19 ± 0.01 ^a^
L2.5	0.18 ± 0.03 ^ab^	0.37 ± 0.08 ^a^	0.10 ± 0.01 ^a^	0.36 ± 0.06 ^a^	n.a.	0.18 ± 0.01 ^a^
L5	0.16 ± 0.02 ^b^	0.42 ± 0.05 ^a^	0.10 ± 0.01 ^a^	0.41 ± 0.11 ^a^	n.a.	0.20 ± 0.01 ^a^
*p-value*	*0.0349*	*0.5160*	*0.2610*	*0.0587*	*-*	*0.1377*

C—control (pasta from semolina); R2.5-5—pasta from semolina flour fortified with 2.5–5% of Reishi mushroom powder; L2.5-5—pasta from semolina flour fortified with 2.5–5% of Lion’s Mane mushroom powder; LOXI—lipoxygenase inhibition; COX2I—cyclooxygenase-2 inhibition; n.a.—no activity. Means with different superscripts within a column are significantly different at *p* ≤ 0.05.

**Table 6:** Anticancer properties of pasta supplemented with dried Reishi and Lion’s Mane mushrooms.

Samples	Anticancer Properties EC50 (mg/mL)	
	GD	GID
C	0.18 ± 0.01 ^a^	0.07 ± 0.01 ^ab^
R2.5	0.15 ± 0.01 ^a^	0.08 ± 0.01 ^a^
R5	0.28 ± 0.01 ^a^	0.08 ± 0.01 ^b^
L2.5	0.16 ± 0.01 ^a^	0.08 ± 0.01 ^ab^
L5	0.20 ± 0.02 ^a^	0.08 ± 0.01 ^ab^
*p-value*	*0.0811*	*0.0255*

C—control (pasta from semolina); R2.5-5—pasta from semolina flour fortified with 2.5–5% of Reishi mushroom powder; L2.5-5—pasta from semolina flour fortified with 2.5–5% of Lion’s Mane mushroom powder; GD—gastric digested samples; GID—gastrointestinal digested samples; AGS—Human Caucasian gastric adenocarcinoma; HT 29—Human Caucasian colon adenocarcinoma. Means with different superscripts within a column are significantly different at *p* ≤ 0.05.

## Data Availability

All relevant data are included in the article.

## Supplementary Materials

The following supporting information can be downloaded at: https://www.mdpi.com/article/10.3390/antiox12030738/s1, Table S1: Parameters of all the molecules monitored with the MRM method; Table S2: Qualitative–quantitative analysis of phenolic compounds using the LC-MS/MS technique in ethanolic (A), PBS (B), and GID (C) extracts.
