# Supplementary material for: The Addition of Reishi and Lion’s Mane Mushroom Powder to Pasta Influences the Content of Bioactive Compounds and the Antioxidant, Potential Anti-Inflammatory, and Anticancer Properties of Pasta
Source: Antioxidants (Basel). 2023 Mar 17;12(3):738. doi: 10.3390/antiox12030738 (PMC10044984; doi:10.3390/antiox12030738)
Supplement: Supplementary file 1 [file antioxidants-12-00738-s001.zip › antioxidants-2211340-supplementary.pdf]

Table S1. Parameters of all the molecules monitored with the MRM method

Compound	Precursor ion Q1 (m/z)	Product ion Q2 (m/z)	CE (V)	Retention time (min)
Gallic acid 1	169	124.9	-20	1.89
Gallic acid 2	169	78.9	-30	1.89
3,4-Dihydroxybenzoic acid 1	153	108.9	-20	3
3,4-Dihydroxybenzoic acid 2	153	65	-26	3
Chlorogenic acid 1	352.9	190.8	-26	3.45
Chlorogenic acid 2	352.9	84.9	-26	3.45
Caffeic acid 1	179	135	-22	3.75
Caffeic acid 2	179	107	-32	3.75
Syringic acid 1	197	181.9	-18	3.8
Syringic acid 2	197	122.9	-30	3.8
Daidzin 1	415.1	251.9	-38	3.96
Daidzin 2	415.1	222.8	-38	3.96
Rutin 1	609	300.3	-46	4.01
Rutin 2	609	270.9	-46	4.01
Ellagic acid 1	301	284	-40	4.27
Ellagic acid 2	301	229	-38	4.27
p-Coumaric acid 1	163	119	-20	4.33
p-Coumaric acid 2	163	92.9	-42	4.33
Salicylic acid 1	137	93	-16	4.46
Salicylic acid 2	137	75	-40	4.46
Vanillin 1	150.9	136.1	-18	4.65
Vanillin 2	150.9	91.9	-26	4.65
Cynarin 1	515	353	-28	4.68
Cynarin 2	515	191	-42	4.68

m-Coumaric acid 1	163	119	-20	4.72
m-Coumaric acid 2	163	92.9	-42	4.72
Ferulic acid 2	193	177.9	-18	4.74
Ferulic acid 1	193	134	-22	4.74
Sinapic acid 1	223.1	164	-22	4.78
Sinapic acid 2	223.1	148.9	-28	4.78
Rosmarinic acid 1	358.9	160.9	-22	4.8
Rosmarinic acid 2	358.9	132.6	-40	4.8
Naringin	579	271	-32	5.03
Quercetin 1	301	121	-34	7.08
Quercetin 2	301.1	150.9	-30	7.08
trans-cinnamic-d7 acid	153.9	110	-16	7.4
trans-cinnamic acid 1	146.9	77	-28	7.53
trans-cinnamic acid 2	146.9	103	-14	7.53
Apigenin 1	269.9	116.9	-42	8.33
Apigenin 2	269.9	150.9	-34	8.33
Genistein 1	269	133	-30	8.42
Genistein 2	269	159	-40	8.42
Naringenin 1	271	150.9	-24	8.51
Naringenin 2	271	118.9	-34	8.51

Table S2. Qualitative-quantitative analysis of phenolic compounds using the LC-MS/MS technique in ethanolic (A), PBS (B) and GID (C)extracts

A (Ethanolic extracts)																
**Name of polyphenolic compound**	[ng/g DW]															
	3,4-Dihydroxybenzoic acid	Caffeic acid	Syringic acid	Daidzin	Rutin	Ellagic acid	*p*-Coumaric acid	Salicylic acid	Vanillin	Ferulic acid	Sinapic acid	Rosmarinic acid	t-Cinnamic acid	Genistein	Naringenin
**C**	13.73±0.12[a]	<1	153.03±2.37[ab]	n.d	<1	n.d	314.90±6.06[ab]	<1	112.17±13.05[a]	1587.57±85.27[a]	177.77±4.56[ab]	n.d	n.d	n.d	n.d

**R2.5**	186.60±7.27^{ab}	19.33±0.92^{a}	862.10±27.37^{ab}	<1	<1	n.d	287.67±10.5^{ab}	<1	105.73±7.5^{a}	1314.03±78.17^{ab}	336.67±18.13^{a}	<1	n.d	n.d	<1
**R5**	316.60±0.69^{b}	35.17±16.22^{a}	1242.43±77.31^{a}	<1	<1	n.d	197.33±5.48^{a}	<1	96.83±6.29^{a}	918.23±20.50^{b}	256.47±4.27^{ab}	<1	n.d	n.d	<1
**L2.5**	22.83±0.92^{ab}	<1	106.43±6.12^{ab}	n.d	<1	n.d	343.83±15.99^{ab}	<1	104.57±2.02^{a}	1436.23±78.35^{ab}	120.50±2.94^{ab}	<1	n.d	n.d	n.d
**L5**	26.37±0.64^{ab}	<1	93.87±2.83^{b}	n.d.	<1	n.d	381.83±8.08^{b}	<1	84.70±5.89^{a}	1457.43±2.66^{ab}	115.87±1.62^{b}	<1	n.d	n.d	n.d
P - value	*0.0086*	*0.0809*	*0.0086*	-	-	-	*0.0086*	-	*0.0651*	*0.0189*	*0.0086*	-	-	-	-

B (PBS extracts)

**Name of polyphenolic compound**	**[ng/g DW]** 3,4-Dihydroxybenzoic acid	Caffeic acid	Syringic acid	Daidzin	Rutin	Ellagic acid	*p*-Coumaric acid	Salicylic acid	Vanillin	Ferulic acid	Sinapic acid	Rosmarinic acid	t-Cinnamic acid	Genistein	Naringenin
**C**	26.33±5.08^{a}	n.d	140.13±16.86^{ab}	n.d	<1	n.d	209.10±16.45^{a}	<1	69.90±11.78^{ab}	1247.77±150.46^{a}	48.57±10.33	n.d	n.d	n.d	n.d
**R2.5**	15.70±3.64^{a}	n.d	727.67±24.31^{ab}	<1	n.d	n.d	187.70±1.04^{a}	<1	70.73±4.62^{ab}	809.20±31.00^{a}	n.d	n.d	n.d	<1	<1
**R5**	30.27±8.54^{a}	n.d	2400.87±900.20^{a}	<1	n.d	n.d	261.80±103.75^{a}	<1	134.83±47.57^{a}	1406.67±595.71^{a}	n.d	n.d	n.d	<1	<1
**L2.5**	n.d	n.d	101.50±6.06^{ab}	n.d	n.d	n.d	251.80±2.42^{a}	<1	65.83±5.31^{ab}	1004.83±1.33^{a}	n.d	n.d	n.d	n.d	n.d
**L5**	n.d	n.d	97.90±0.17^{b}	n.d.	n.d	n.d	277.73±2.54^{a}	<1	57.33±5.77^{b}	1159.57±15.13^{a}	n.d	n.d	n.d	n.d	n.d
P - value	*0.5660*	--	*0.0097*	-	-	-	*0.1088*	-	*0.0349*	*0.1416*	-	-	-	-	-

C (GID samples)

**Name of polyphenolic compound**	**[ng/g DW]** 3,4-Dihydroxybenzoic acid	Caffeic acid	Syringic acid	Daidzin	Rutin	Ellagic acid	*p*-Coumaric acid	Salicylic acid	Vanillin	Ferulic acid	Sinapic acid	Rosmarinic acid	t-Cinnamic acid	Genistein	Naringenin
**C**	n.d	n.d	124.85±0.52^{b}	n.d	<1	n.d	143.08±2.19^{a}	<1	179.85±7.09^{a}	936.87±43.13^{a}	68.28±0.09^{b}	<1	n.d	<1	n.d
**R2.5**	32.27±0.79^{a}	n.d	502.22±49.95^{ab}	n.d	<1	n.d	159.70±12.07^{a}	<1	202.83±16.88^{a}	906.57±73.13^{a}	136.01±8.49^{a}	n.d	n.d	<1	<1

**R5**	26.62±03.85^{a}	n.d	799.85±32.05^{a}	n.d	<1	n.d	131.31±2.45^{a}	<1	188.94±0.52^{a}	760.00±30.70^{a}	135.71±3.50^{ab}	n.d	n.d	<1	<1
**L2.5**	n.d	n.d	109.19±16.62^{ab}	n.d	n.d	n.d	153.89±20.56^{a}	<1	223.28±43.65^{a}	863.94±121.77^{a}	23.23±5.16^{b}	n.d	n.d	<1	n.d
**L5**	n.d	n.d	100.30±0.52^{b}	n.d	n.d	n.d	159.75±9.62^{a}	<1	207.47±36.39^{a}	845.40±42.60^{a}	31.16±4.29^{ab}	n.d	n.d	<1	n.d
P - value	*0.0809*	-	*0.0112*	-	-	-	*0.0746*	-	*0.5257*	*0.1309*	*0.0122*	-	-	-	-
